# High-Quality Draft Genome Sequences of Two *Xanthomonas citri* pv. malvacearum Strains

**DOI:** 10.1128/genomeA.00674-13

**Published:** 2013-08-29

**Authors:** Sébastien Cunnac, Stéphanie Bolot, Natalia Forero Serna, Erika Ortiz, Boris Szurek, Laurent D. Noël, Matthieu Arlat, Marie-Agnès Jacques, Lionel Gagnevin, Sébastien Carrere, Michel Nicole, Ralf Koebnik

**Affiliations:** UMR 186 IRD-Cirad-Université Montpellier 2 “Résistance des Plantes aux Bioaggresseurs,” Montpellier, Francea; INRA, Laboratoire des Interactions Plantes Micro-organismes (LIPM), Castanet-Tolosan, Franceb; CNRS, Laboratoire des Interactions Plantes Micro-organismes (LIPM), Castanet-Tolosan, Francec; Université de Toulouse, Université Paul Sabatier, Toulouse, Franced; INRA, Institut de Recherche en Horticulture et Semences (IRHS), Beaucouzé, Francee; UMR “Peuplements Végétaux et Bioagresseurs en Milieu Tropical” (PVBMT), La Réunion, Francef

## Abstract

We report high-quality draft genome sequences of two strains (race 18 and 20) of *Xanthomonas citri* pv. malvacearum, the causal agent of bacterial blight of cotton. Comparative genomics will help to decipher mechanisms provoking disease and triggering defense responses and to develop new molecular tools for epidemiological surveillance.

## GENOME ANNOUNCEMENT

*Xanthomonas citri* pv. malvacearum is the causal agent of bacterial blight (BB), one of the most devastating diseases of cotton (*Gossypium* spp.). BB of cotton was first reported in the United States by Atkinson, who described several symptoms, such as angular leaf spots, water-soaked lesions, stem black arm, boll rot, and plantlet burning (1). In the second half of the 20th century, BB became a limiting factor of fiber production in all major cotton-producing regions of Africa, Asia, Australia, and North America (2). The use of resistant cultivars is usually the most efficient way to manage disease. However, resistance was repeatedly overcome by the appearance of new races. More than 20 races have been described, including highly virulent strains that were isolated in Central Africa in the 1980s (2). To better understand the molecular basis of provoking disease and of triggering defense responses and to develop new molecular markers for epidemiological surveillance, we sequenced the genomes of two strains originating from Burkina Faso and belonging to the highly virulent race 20 and the less virulent race 18 (3).

Both strains were sequenced using the Illumina Hi-Seq2000 platform (GATC Biotech, Germany). The shotgun sequencing yielded 40,654,599 read pairs (29,066,551 100-bp paired-end reads with an insert size of 250 bp and 11,588,048 50-bp mate-pair reads with an insert size of 3 kb) and 43,359,649 read pairs (19,340,592 100-bp paired-end reads and 24,019,057 50-bp mate-pair reads) for strains X18 and X20, respectively. A combination of Velvet (4), SOAPdenovo, and SOAPGapCloser (5) yielded 22 contigs larger than 500 bp (*N*_50_, 705,301 bp), with the largest contig of 2,062 kb, for a total assembly size of 4,989,917 bp for strain X18 and 17 contigs larger than 500 bp (*N*_50_, 1,006,603 bp), with the largest scaffold of 1,770 kb, for a total assembly size of 5,216,199 bp for strain X20.

Multilocus sequence analyses of six housekeeping genes described earlier for xanthomonads confirmed that strains X18 and X20 belong to *X. citri* pv. malvacearum (2,745 bp with 100% identity) (6), corresponding to DNA-DNA homology group 9.5, which also includes *X. citri* pv. citri (2,730 identical residues) and *X. citri* pv. glycines (2,729 identical residues) (7). The genome encodes a canonical type III protein secretion system (8) and several type III effectors, including transcriptional activator-like (TAL) effectors, which are responsible for symptom formation and avirulence reactions on cotton (9). The availability of two genome sequences of *X. citri* pv. malvacearum will critically aid in developing new molecular typing tools for epidemiological surveillance and guiding breeding programs based on rapid and accurate identification of predominant lineages.

### Nucleotide sequence accession numbers. 

These whole-genome shotgun projects have been deposited in GenBank under the accession numbers ATMA00000000 for strain X18 and ATMB00000000 for strain X20.
